# Challenges of breastfeeding during COVID-19 and baby friendly protocols adopted at a maternity health center in the northern Emirates of UAE: a comprehensive review

**DOI:** 10.1186/s41043-024-00544-0

**Published:** 2024-05-14

**Authors:** Hafiz Ahmad, Ghania Shehzad Qureshi, Luma Bassam Odeh, Lovely M. Annamma, Ashfaque Hossain, Azhar Khan, Shabirul Haque, Aswan Kinan Rasheed

**Affiliations:** 1 Department of Medical Microbiology and Immunology, RAK College of Medical Sciences, RAK Medical & Health Sciences University, 241, RAKMHSU Campus, Ras Al Khaimah, UAE; 2 Microbiology and Molecular Division, NGS & COVID Laboratory, RAK Hospital, Ras Al Khaimah, UAE; 3 RAK College of Medical Sciences, RAK Medical & Health Sciences University, Ras Al Khaimah, UAE; 4 Department of Clinical Sciences, College of Dentistry, Ajman University, Ajman, UAE; 5 Faculty of Biotechnology and Applied Sciences, Shoolini University of Biotechnology and Management Sciences, Solan, India; 6 The Feinstein Institute of Medical Research, Northwell Health, Manhasset, NY USA; 7 International Board-Certified Lactation Consultant, Al-Nakheel Public Health Clinic, Emirates Health Services, Ministry of Health and Prevention (MOHAP), Ras Al Khaimah, UAE

**Keywords:** COVID-19, Breastfeeding, SARS-CoV-2 emerging variants, Vaccination, Hygiene recommendations, Neonates, Literature Review, UAE

## Abstract

**Background:**

The outbreak of Coronavirus disease (COVID-19), caused by Severe Acute Respiratory Syndrome Coronavirus 2 (SARS CoV-2) has caused worldwide panic in the global population taking people's lives, creating fear, and affecting mother–child relationships. Many questions were raised on the dangers of being infected with COVID-19 for newborns and safety concerns during feeding by COVID-19-positive mothers. Moreover, questions and doubts about the safety of the administration of vaccinations for nursing mothers are still open. This review attempts to fill the existing literature gap by exploring concepts concerning COVID-19 and breastfeeding mothers, the safety of vaccinations, the beneficial effects of breastfeeding on both mother and child, important hygiene recommendations for SARS-CoV-2 infected mothers, and possible solutions to optimize breastfeeding and safety precautions amidst the fear of emergence of novel variants.

**Methods:**

All relevant publications from Google Scholar, PubMed, Web of Science, and Scopus search engines from December 2019 to October 2022 related to SARS-CoV-2, breastfeeding, COVID-19, lactating guidelines, and vaccination were included using ‘Breastfeeding AND vaccine AND SARS-CoV-2’ as MESH TERMS. Apart from the literature review, existing maternity protocols followed in Northern UAE were gathered from lactation consultants practicing in the UAE.

**Results:**

Out of 19,391 records generated, only 24 studies were analyzed and summarized in this exhaustive review using the Preferred Reporting Items for Systematic Reviews and Meta-Analyses (PRISMA) flow chart. Previous studies suggest that breastmilk is predominantly the primary source of nutrition for neonates. Breast milk is a rich source of antibodies that help the baby to fight against infections including other benefits. Hygiene recommendations for suspected or confirmed COVID-19-infected mothers are required along with psychological and emotional support.

**Conclusions:**

The administration of vaccinations should be advised and encouraged to protect the mothers with antibodies and the neonates by the passive transmission of antibodies through breast milk. This is a significant reason for not stopping breastfeeding even in case of COVID-19 infection. With adherence to proper hygiene methods, breastfeeding is recommended to be continued as the benefits greatly outweigh the risks.

## Background

The novel coronavirus (SARS-CoV-2) which causes COVID-19 originated in Wuhan; China caused respiratory infections ranging from a common cold to severe respiratory distress. COVID-19 strains spread from person to person through contaminated droplets through sneezing or coughing. Vulnerable populations like infants have been shown to display milder symptoms of the disease similar to a regular cold unless presented with other comorbidities. The most susceptible populations that have been known to develop severe symptoms are the elderly, immunocompromised patients, and individuals with chronic health conditions [1].

Lactating mothers face confusion on whether or not breastfeeding is safe and may be possible during the current pandemic. Breast milk supplements are not a substitute for breast milk [2]. Breastmilk supplies all the energy and nutrients that the infant needs in the first months of life and continues to provide up to half or more of the child's nutritional needs in the second half of the first year and up to one-third in the second year of life. Breastfed children perform better intelligence tests, are less likely to be overweight or obese, and are less likely to have diabetes later in life. Women who breastfeed also have a reduced risk of breast and ovarian cancer [3]. The benefits of breastfeeding greatly outweigh the risks and breastfeeding should be continued whilst following hygiene precautions. With the emergence of several new variants, there are debates on whether nursing mothers should be vaccinated to protect themselves from the virus and whether it adversely affects the breastfed infant This review aims to provide an exhaustive, comprehensive summary of the topic and the experiences of lactation consultants and local experts on handling breastfeeding challenges across local maternity clinics and maternity Ministry of Health hospitals in the northern state of Ras al Khaimah, UAE.

## Methods

**Fig. 1:**
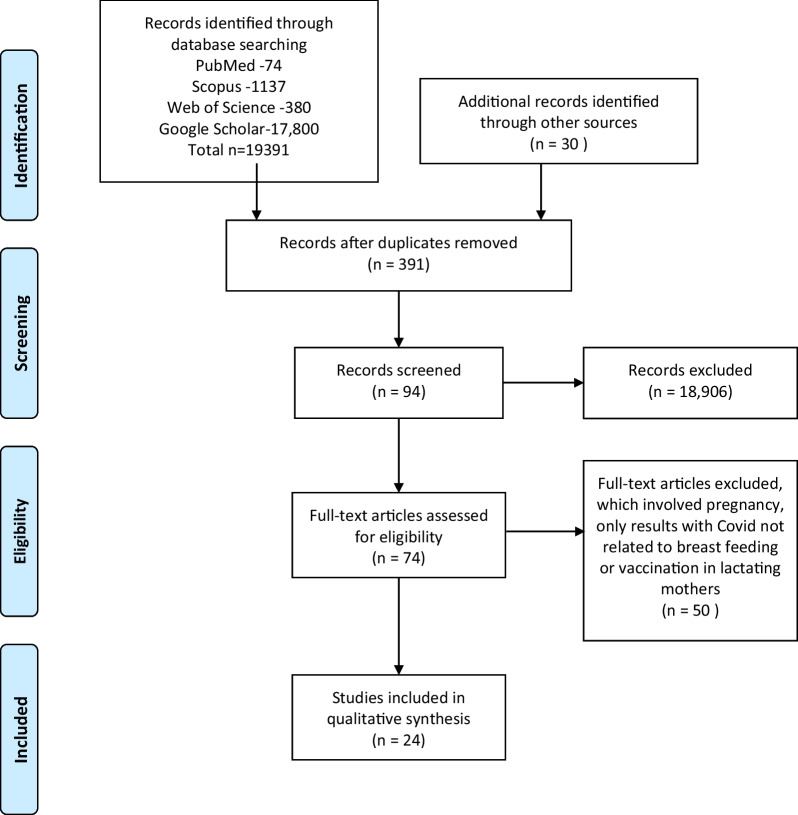
PRISMA flowchart

A literature search was conducted for two years, from January 2020 to October 2022 using ‘Breastfeeding AND vaccine AND SARS-COV-2’ as MESH TERMS to generate a PRISMA flow chart (Fig. 1). A total of 19,391 records were generated, out of which 391 were excluded due to duplication. Various combinations of keywords related to breastfeeding, COVID-19, SARS-CoV2 variants, vaccinations, hygiene recommendations, and lactating guidelines were utilized. Official documents available in English were included. These included published and in-press clinical research articles, as well as interim guides, expert reviews, or guidelines/official statement documents from international associations. A total of twenty-four studies were finally analyzed and summarized in this exhaustive review using the Preferred Reporting Items for Systematic Reviews and Meta-Analyses (PRISMA) flow chart. The study was approved by the RAK Medical & Health Sciences University Research and Ethics Committee (RAKMHSU-REC-036-2020/2021-UG-M).

## Discussion (Table 1)

**Table 1:** Included studies

References	Title	Type of study	Outcome
Perl SH, Uzan-Yulzari A, Klainer H, Asiskovich L, Youngster M, Rinott E, Youngster I [28]	SARS-CoV-2-specific antibodies in breast milk after COVID-19 vaccination of breastfeeding women	Prospective study	SARS-CoV-2-specific IgA and IgG antibodies in breast milk for 6 weeks after vaccination. IgA secretion was evident at 2 weeks after vaccination followed by a spike in IgG after 4 weeks (a week after the second vaccine)
Garg I, Shekhar R, Sheikh AB, Pal S. [30]	COVID-19 Vaccine in pregnant and lactating women: a review of existing evidence and practice guidelines	Review	COVID-19 vaccines can be given to breastfeeding individuals who meet the criteria for vaccination based on ACIP- (Advisory Committee on Immunization Practices)
McLaurin-Jiang S, Garner CD, Krutsch K, Hale TW. [32]	Maternal and child symptoms following covid-19 vaccination among breastfeeding mothers	Cross-sectional survey	COVID-19 vaccination caused minimal disruption of lactation or adverse impact on the breastfed child
Charepe N, Gonçalves J, Juliano AM, Lopes DG, Canhão H, Soares H, Serrano EF [10]	COVID-19 mRNA vaccine and antibody response in lactating women: a prospective cohort study	Prospective cohort study	The presence of antibodies in human milk is reported, but immunization through breastfeeding is yet to be established
Bertrand K, Honerkamp-Smith G, Chambers CD [39]	Maternal and child outcomes reported by breastfeeding women following messenger RNA COVID-19 vaccination	Prospective cohort study	All mRNA COVID-19 vaccines are safe for lactating mothers
Pérez-Bermejo M, Peris-Ochando B, Murillo-Llorente MT 2021 Martins I, Louwen F, Ayres-de-Campos D, Mahmood T. [40]	COVID-19: relationship and impact on breastfeeding-a systematic review European board of obstetrics and gynec (EBOG)position statement on COVID-19 vaccination for pregnant and breastfeeding women	Systematic Review	Direct breastfeeding and maintaining appropriate protective measures should be encouraged
Muyldermans J, De Weerdt L, De Brabandere L, Maertens K, Tommelein E. [25]	The effects of COVID-19 vaccination on lactating women: a systematic review of the literature	Systematic review	There is evidence that the administration of a COVID-19 vaccine is safe and poses no additional risk to the breastfeeding woman or the breastfed baby
Narayanaswamy V, Pentecost BT, Schoen CN, Alfandari D, Schneider SS, Baker R, Arcaro KF [15]	Neutralizing antibodies and cytokines in breast milk after coronavirus disease 2019 (COVID-19) mRNA vaccination	A cohort study on 30 lactating mothers	Elevation of interferon-γ. The immune response to maternal vaccination was reflected in breastfed infants: anti-RBD IgG and anti-RBD IgA were detected in 33% and 30% of infant stool samples (Passive immunity against Covid -19
Pace RM, Williams JE, Järvinen KM, Belfort MB, Pace CDW, Lackey KA, Gogel AC, Nguyen-Contant P, Kanagaiah P, Fitzgerald T, Ferri R, Young B, Rosen-Carole C, Diaz N, Meehan CL, Caffé B, Sangster MY, Topham D, McGuire MA, Seppo A, McGuire MK [16]	Characterization of SARS-CoV-2 RNA, antibodies, and neutralizing capacity in milk produced by women with COVID-19	In Vitro study	62% of the milk samples were able to neutralize SARS-CoV-2 whereas milk samples collected before the COVID-19 pandemic were unable to do so
Lechosa-Muñiz C, Paz-Zulueta M, Mendez-Legaza JM, Irure-Ventura J, Cuesta González R, Calvo Montes J, López-Hoyos M, Llorca J, Cabero-Pérez MJ [37]	Induction of SARS-CoV-2-specific IgG and IgA in serum and milk with different SARS-CoV-2 vaccines in breastfeeding women: a cross-sectional study in northern Spain. I	Clinical trials	Vaccines given to breastfeeding mothers offer their infants IgA and IgG isotype antibodies directed against SARS-CoV-2 protein S in breast milk
Lamers M, van der Mijle A, van Hunsel F, de Vries L, van Puijenbroek E, Ceulemans M [41]	COVID-19 vaccination during breastfeeding and its possible negative effect on milk production and supply: a preliminary observation	Observational study	occurrence of reduced milk supply appears to be low and transient,
Pang Z, Hu R, Tian L, Lou F, Chen Y, Wang S, He S, Zhu S, An X, Song L, Liu F, Tong Y, Fan H [6]	Overview of breastfeeding under COVID-19 pandemic	Overview	Breastfeeding is to be continued with recommendations and guidance for breastfeeding set by international organizations
Rosenberg-Friedman M, Kigel A, Bahar Y, Werbner M, Alter J, Yogev Y, Dror Y, Lubetzky R, Dessau M, Gal-Tanamy M, Many A, Wine Y [53]	BNT162b2 mRNA vaccine-elicited antibody response in blood and milk of breastfeeding women	Prospective study	The response in breast milk includes both IgG and IgA with neutralization capacity
Whited N, Cervantes J [27]	Antibodies against SARS-CoV-2 in human breast milk after vaccination: a systematic review and meta-analysis	Systematic review	of high rates of positivity for antibodies in breast milk following COVID-19 immunizations
Olearo F, Radmanesh LS, Felber N, von Possel R, Emmerich P, Pekarek N, Pfefferle S, Nörz D, Hansen G, Diemert A, Aepfelbacher M, Hecher K, Lütgehetmann M, Arck PC, Tallarek AC [42]	Anti-SARS-CoV-2 antibodies in breast milk during lactation after infection or vaccination: a cohort study	Cohort Study	Breast milk antibodies in all groups showed neutralization capacities against an early pandemic SARS-CoV-2 isolate (HH-1) and Omicron variant, although with lower antibody titer
Mulleners SJ, Juncker HG, van Gils MJ, van Goudoever JB, van Keulen BJ [31]	Human milk antibody response after combining two different COVID-19 vaccines: mix-and-match	Clinical trials	heterologous vaccination with AZD1222 and an mRNA-based vaccine can elicit a significant SARS-CoV-2 specific IgA response in human milk
Perez SE, Luna Centeno LD, Cheng WA, Marentes Ruiz CJ, Lee Y, Congrave-Wilson Z, Powell RL, Stellwagen L, Pannaraj PS [26]	Human milk SARS-CoV-2 antibodies up to 6 months after vaccination	Prospective longitudinal study	The data suggest that human milk SARS-CoV-2-specific antibodies may be available to milk-fed infants for up to 6 months
Abuidhail J, Tamim F, Abdelrahman RY, Al-Shalabi E [59]	Knowledge and practices of breastfeeding mothers towards prevention of the emerging coronavirus (COVID-19)	Cross-sectional study	Participants have basic knowledge about COVID-19 in general, but they have improper preventive breastfeeding practices against the disease
Mosalli R, Paes B [17]	Setting realistic goals for feeding infants when their mothers have suspected or confirmed COVID-19	Review	parents should fully understand the current evidence, availability of personnel to support breastfeeding, protective spaces, caseloads, and resources available to implement existing recommendations
Peroni DG, Fanos V [4]	Lactoferrin is an important factor when breastfeeding and COVID-19 are considered	Review	we believe that early breastfeeding provides vital prevention during viral epidemics, due to the high value of colostrum and breast milk and the specific role of lactoferrin
Vazquez-Vazquez A, Dib S, Rougeaux E, Wells JC, Fewtrell MS [73]	The impact of the COVID-19 lockdown on the experiences and feeding practices of new mothers in the UK: Preliminary data from the COVID-19 new mum study	Data review	Lockdown has impacted maternal experiences, resulting in distress for many women
Low JM, Low YW, Zhong Y, Lee CYC, Chan M, Ng NBH, Amin Z, Ng YPM. [29]	Titers and neutralizing capacity of SARS-CoV-2-specific antibodies in human milk: a systematic review	Systematic review	Human milk of lactating individuals after COVID-19 infection contains anti-SARS-CoV-2-specific IgG, IgM, and/or IgA, even after mild or asymptomatic infection
Davanzo R [60]	Breastfeeding at the time of COVID-19: do not forget expressed mother's milk, please	Review	Breastfeeding at the time of COVID-19: do not forget expressed mother's milk
Walker KF, O'Donoghue K, Grace N, Dorling J, Comeau JL, Li W, Thornton JG [69]	Maternal transmission of SARS-COV-2 to the neonate, and possible routes for such transmission: a systematic review and critical analysis	systematic review and critical analysis	wear a fluid-resistant surgical face mask, if available while feeding or caring for the baby

### Immunity against diseases

The main advantage of breastfeeding is providing immunity to fight against diseases. Breastfeeding offers natural milk which is the most preferred and nutritionally enriched source of food for the newborn. Breastmilk is particularly essential in reducing infectious conditions, with its anti-infective and anti-inflammatory factors, especially common cold, Influenza, and allergic conditions. Even if a mother is sick or infected, breast milk can provide the necessary antibodies against that specific infection [3, 4].

The pandemic has thrown upon challenges of logistics, shortages or panic purchasing, baby formula, bottles, and other feeding supplies were not readily available especially in developing countries, leading to errors in the preparation of baby formulas that occur at any time, particularly during pandemic chaos. Despite a pandemic, breastfeeding allows the mother to provide her child with independent total food solutions at her convenience. The inappropriate marketing of breast milk substitutes, bottles, and treats during this time of pandemic increases the risks of child mortality, morbidity, and malnutrition [5, 6]. The goal of disaster relief for infants and the best way to save vulnerable infants must therefore be to promote lactation and to help women breastfeed [7, 8].

These antibodies are present in high amounts in the colostrum. Colostrum contains high amounts of peptides and β-defensin-2 levels in breastmilk which is a defense molecule for intestinal health in pediatric patients [9, 10]. Other factors in breastmilk directly stimulate and promote the immune system, such factors include lactoferrin and interleukin-6, -8, and -10. These are proteins that help balance the inflammatory response of the immune system, which is needed for immune function but can be harmful in excess [11]. Past and current literature also supports that there is evidence that human milk is a rich source of extracellular vesicles (EVs) equipped with immuno-modulatory properties [12].

### Innate immunity and COVID-19

After birth, maternal antibodies protect the infant from any previous microbes that the mother might have encountered throughout her life. The mother's milk transfers the immune cells such as -macrophages, neutrophils, and lymphocytes to the infants to protect them from various diseases and build up their innate immunity [13].

Although vaccines are now available to protect people from potentially lethal diseases, all germs are new to children. Frequent infections occurring in the first years of life allow the body to build a memory of T and B memory cells that prevent the re-infection or development of disease by pathogens that are commonly encountered. The pediatric immune system is therefore prepared and fit to respond to new pathogens, a function that may be compromised in adults and diminished in elderly people aged 70 years and older. The children's immune preparedness for new pathogens could be dependent on a variety of factors, including SARS-CoV-2. First, natural antibodies play the most key role in the initial stages of infection. Natural antibodies, mostly IgM isotype, have a wide reactivity and variable similarity. They can produce high-affinity antibodies and memory B cells that will clear the virus and prevent re-infection. High-affinity antibodies are expressed as switched memory B cells (MBCs). In humans, natural antibodies are produced by innate or IgM MBCs, a population of MBCs that is produced separately from germ centers and is most commonly found in children. In addition to the production of antibodies, B cells also have the function of secreting cytokines. IL-10, a potent anti-inflammatory cytokine, is produced by neonatal B cells, activated B cells [13], and plasma IgA cells. Thus, the child's immune response may have the dual function of protecting and reducing immune-mediated tissue damage, particularly in the lungs [14]. In a recently published paper, it was observed that T cells play a more significant role in controlling COVID-19 than other immune cells, and therefore along with measurement of neutralizing antibodies that are known to offer protection, it is important to know the T cell count. In a recent study, the breastmilk before and after COVID-19 infection found that there was an increase in the percentage of macrophages expressing INF-alpha and this signifies the role of macrophages as the main component in the innate immunity of the milk [15].

### COVID-19 and viral transmission through breastfeeding

COVID-19 is a highly infectious disease that only spreads through respiratory droplets. According to ongoing research, breast milk is completely safe and cannot spread the virus, but the baby is still at risk for the disease through droplet infection from the mother [16] If the mother were to cough or sneeze near the child, and the child inhaled those droplets, this could lead to infection. Furthermore, if the child were to touch a previously contaminated surface and touch their nose, eyes, or mouth- that could lead to infection as well. Hence, for the protection of the child, the mother should follow proper precautions such as wearing a mask while breastfeeding, washing hands frequently with soap and water for 20 s (or using an alcohol-based rub), regularly disinfecting surfaces, etc. [17].

### Effects of COVID-19 variants on the transmission of the virus and disease severity

The rapid evolution of the virus and the increase in SARS-CoV-2 infectivity gives rise to the emergence of novel variants. Therefore, understanding the mechanism of its pathogenicity and virulence, as well as developing effective therapeutic strategies is vital.

More recently, the emergence of a strain with a deletion of nine nucleotides in the nsp1 gene (nucleotides 686–694 corresponding to amino acids 241–243) was identified. It was suggested by structural analysis that this deletion could potentially affect the C-terminal region of the protein that plays a role in the regulation of viral replication, besides harming the host's gene expression [18]. The following studies confirmed these results and highlighted that SARS-CoV-2 is undergoing profound genomic changes [19, 20]. While the D614G mutation increases SARS-CoV-2 virulence [21]. The precise biology of the other mutations is still unknown. However, nsp1, which is the leader protein, plays a vital role in the inhibition of the host’s innate immunity, in particular the expression of interferon-alpha [22], which is probably the most important determinant of viral pathogenicity. The specific effects of each virus particularly on nursing mothers and their breastfed babies are still unknown and more research needs to be done in this area.

### COVID-19 vaccination in breastfeeding mothers

A sharp upturn in infections due to the delta and omicron variants and a slowdown in vaccinations have pushed governments to make COVID-19 shots mandatory for health workers and other high-risk groups. Although the effects of the different variants on breastfeeding mothers and the transmission of the newer variants through breastmilk are still unknown, as per existing literature approved COVID-19 vaccines are expected to provide some degree of protection against emerging new virus variants because these vaccines elicit a broad immune response involving a range of antibodies and T-cells. Therefore, changes or mutations in the virus should not make vaccines completely ineffective. As a result, mothers are advised to take the SARS-CoV2 vaccine to protect themselves and their newborns through antibodies [23–25]. Moreover, a study suggests that human milk SARS-CoV-2-specific antibodies may be available to milk-fed infants for up to 6 months [26–28]. In addition, donor milk from vaccinated mothers retains IgG and neutralizing activity [29]. Everyone should get vaccinated to attain herd immunity and decrease the severity of the virus in case anyone should be infected. The president of the OBG Society of Emirates Medical Association, UAE as per the evidence-based literature stated that “Clinical studies show that the mRNA vaccine is safe for women who are breastfeeding or planning to conceive unless the patient has certain medical contraindications to vaccines or any vaccine component [30]. Mothers who are breastfeeding can take an mRNA vaccine as per the latest DHA vaccine guidelines. There is no need to stop breastfeeding before or after vaccination as scientific evidence after mixing different vaccinations has shown antibody protection in milk [31, 32]. Though antibodies are present immunization through breastfeeding is not confirmed [10].

There are four types of vaccines in clinical trials: whole inactivated virus, protein subunit, viral vector, and nucleic acid. All four types of vaccines work in diverse ways but work to achieve the same goal: building immunity against SARS-COV19. Additionally, a study showed that COVID-19 vaccination among breastfeeding mothers resulted in minimal disruption of lactation or adverse impact on the breastfed child [33]. In the USA, the first vaccine to get approved on an emergency basis was the Pfizer/BioNTech mRNA Vaccine. Soon after, Moderna mRNA Vaccine was approved followed by the Johnson & Johnson human adenovirus vaccine on February 26, 2021.

A study was conducted on lactating mothers whose breastmilk was evaluated after the second dose of the mRNA and vaccines. All samples were shown to have IgG antibodies specific to SARS-CoV 2 spike protein [31, 34].

For instance, mRNA-based vaccines include mRNA for the SARS-CoV-2 spike protein encased in lipid nanoparticles where the mRNA sequence only encodes this protein. These particles are injected into the muscle, where the lipid nanoparticles are taken up by muscle cells. These muscle cells then transcribe the mRNA to produce spike protein. The spike protein made by the cell stimulates an immune response, protecting the individual from COVID-19 illness [35]. It is highly unlikely that the vaccine lipid nanoparticles would enter the bloodstream and reach breast tissue. In case it does, it is even less likely that either the intact nanoparticle or mRNA would transfer into the breast milk. In the unlikely event that mRNA is present in milk, it would be expected to be digested by the child and would not have any biological effects. While there is little plausible risk for the child, there is a biologically plausible benefit [36]. Neither inactivated nor live vaccines administered to a lactating woman affect the safety of breastfeeding for women or their infants. Breastfeeding does not adversely affect immunization and is not a contraindication for any vaccine, except for the smallpox vaccine [37–39]. Hence the conclusion from all studies was vaccination is safe and should be advocated for pregnant women [40]. Lamers et al. [41] in an observational study reported that after vaccination the milk supply was low. Some studies also observed that booster vaccination can help protect the mother and newborn [42].

### Challenges of breastfeeding during COVID-19 duration

According to multiple world-renowned medical institutes and universities such as WHO [43], and CDC [44], the main challenge that breastfeeding mothers are facing is uncertainty in providing breast milk to the neonates in fear that the virus may infect the child. Apprehension of these consequences has no scientific basis since sources have explicitly stated that according to recent research, breastmilk does not contain the SARS-CoV-2 viroid and is highly recommended for a newborn baby [17]. It is the responsibility of healthcare workers to educate the general population on this matter.

In a case reported in China and Germany, it was found that the virus was present in two SARS-CoV-2-positive patients’ milk [45, 46]. In the report, the infants tested positive as well. However, both infants were exposed to their infected mothers and the contaminated environment. Therefore, it is impossible to determine if breastmilk was the source. If a risk–benefit analysis is performed, the benefits highly outweigh the risks and therefore, it is best to continue breastfeeding [47]. In case the mother is not too sick to nurse the child, due to confirmed or suspected cases of COVID-19, it is best to provide the child with other sources of breastmilk. This can be in the form of expressed milk or donated breast milk. If they are not available, then wet nursing can be considered. As a last option, formula milk can be considered as well. However, it is important to note that human milk should be prioritized [48].

According to research done at the University of Alberta, another challenge that new nursing mothers face during these times is depression, hesitancy, and stress. Lactating mothers are globally facing prenatal and postnatal anxiety which is not only negatively impacting breastfeeding duration but also increasing hormones such as cortisol, cytokines, and serotonin which directly affect neonates[49]. During this pandemic, some mothers were forced to go through labor and birth alone while some hospitals were not allowing parents and families to visit their infants and even discharging the mothers early from the hospitals due to a limited amount of time. The families were not given any technical knowledge about lactation care, and nursing of the infant which has negatively impacted the growth and care of the newborns [50, 51].

### Risk of breastfeeding in COVID-positive patients

A case series of 22 breastfeeding mothers with COVID-19 infection were followed up for 1.8 months. During follow-up, it was observed that no neonates were infected. This indicates that breastfeeding is safe provided all the necessary hygiene measures are taken [52].

### Transmission of SARS-CoV2 to the newborn by breastfeeding

Since new strains of SARS-CoV-2 are emerging rapidly, it is vital to study their transmission in human milk. Detection of SARS-CoV-2 specific IgG and IgM antibodies was documented in blood samples of some neonates [53]. IgG is an antibody with a smaller molecular weight and structure, hence it can be passively transferred across the placenta to the fetus, whereas IgM cannot be readily transferred through the placenta due to its large macromolecular structure. Therefore, scientists are predicting the presence of the IgM antibody in the newborn blood sample is due to its transmission through breast milk[54]. Another hypothesis is that since the viral nucleic acid was found in the blood of COVID-19-infected patients, there could be a possibility of intrauterine transfer as well [55]. Although IgM might be transferred via breast milk, there is currently no direct evidence to suggest that SARS-CoV-2 can be transmitted through breast milk [56].

### Breastfeeding practices in the initial stages of the COVID-19 pandemic

In the preliminary stages in China, the use of formula milk for infants born to COVID-19-infected mothers was supported by a few pediatricians. They discouraged direct breastfeeding to eliminate any possibilities of virus transmission to the newborn. Discouraging breastfeeding, however, directly diminishes mother-infant bonding and relationship [57]. Therefore, lactating mothers should follow guidelines during breastfeeding to prevent any kind of infection. The equipment used by the mothers for expressing the milk should not be shared and should be washed and rinsed with cold water followed by rinsing with hot water and soap to maintain proper hygiene [58]. A survey conducted by Jamila et al. [59] indicated a lack of proper knowledge regarding breastfeeding during the coronavirus crisis time was observed.

### SARS-CoV-2 positive mothers and breastfeeding

If a mother is COVID-19 positive it's recommended to encourage breastfeeding by practicing safety precautions of respiratory hygiene. The following guidelines are recommended wearing a mask while breastfeeding and washing hands before and after touching the baby for 20–30 s [60]. In the case of a sick mother, expressed breast milk can be given. The following guidelines are recommended for expressing the milk. A dedicated breast pump can be used to express the milk in a clean protected container held by a person in protective clothing so that there are no chances of the spread of the virus through the container this milk can be stored and frozen for later use [61]. It has also been advised to wipe the bottles with a virucidal agent or diluted bleach (sodium hypochlorite) and these bottles are to be separately stored in the refrigerator [62].

Any infant being breastfed by a suspected or confirmed COVID-19 mother should be considered a suspected COVID-19 case—when the infant's testing results are not available for the duration of the mother's recommended period of home isolation and 14 days thereafter. The same approach should be taken for an infant who has been in contact with any suspected or confirmed case other than the mother. Mothers should inform the healthcare provider for the child that their child has had high-risk contact with a person suspected or confirmed to have COVID-19 [44].

### Advice for COVID-19-positive mothers residing in the same room as their infants?

The Chinese experts recommended the separation of a newborn from the mother of suspected or confirmed COVID-19, but the latest WHO guidelines do not encourage mother and child separation as long as adherence to protocols and guidelines is maintained. A reasonable distance should be kept between the mother and the child whenever possible. It is strongly recommended to wear a cloth face covering and wash their hands whenever she directly cares for the baby [63]. The mother must continue taking these precautions until she is fever-free for 24 h without taking any fever medicines (acetaminophen or ibuprofen); at least 10 days have passed since the COVID-19 symptoms first started, and all the symptoms have improved [63].

### Breastfeeding recommendations for COVID-19-positive mothers with severe infection

In the case of mothers with severe COVID-19 infection, expressed mother's milk consideration is recommended as a choice, maintaining the benefits and nutrition in the mother's milk in the absence of direct breastfeeding [64]. This is also recommended by WHO, the United Nations Children’s Fund, the CDC, the Royal College of Obstetricians and Gynecologists, the International Society of Ultrasound in Obstetrics and Gynecology (ISUOG), the Italian National Institute of Health and Academy of Breastfeeding Medicine. Mayo Clinic also recommends the following guidelines for lactating mothers, "If the milk is to be pumped, hands should be washed before touching any pump or bottle part, with proper pump cleaning. If possible, someone who is not infected should feed the baby the expressed breastmilk."

According to WHO, "The COVID-19 virus has not been detected in the breast milk of any mother with confirmed and suspected COVID-19 and there is currently no evidence that the virus can be transmitted through breastfeeding." Further, it is anticipated that the information and guidance available currently might change in the future due to the availability of more information about the COVID‐19 pandemic, its perinatal transmission, and clinical characteristics of cases of infants born to SARS‐CoV‐2 infected mothers [65].

A mother with suspected or confirmed COVID-19 should be guided to take all possible precautions to avoid spreading the virus to her baby but she should not stop breastfeeding.

### Hygiene recommendations for a breastfeeding mother with a baby suspected of COVID-19 infection

The mother is instructed to wash her hands utilizing soap and water, particularly if her hands are dirty, for at least 20 s, before contacting the baby [66]. If soap and water are not accessible, she should use a hand sanitizer with at least 60% alcohol. In the case when the baby is COVID-19-positive, mothers should wear a proper N-95 mask and change their gown after the breastfeeding cycle. If possible, a shower would be best to prevent infection from the mother to other children who may be susceptible to Infection. But this process can be particularly challenging as the baby may be breastfed several times a day which can make it difficult for the mother. Regardless of whether the milk is being expressed by hand or breast pump, all the previously mentioned precautions must be followed diligently. Mothers should be instructed about recommendations on the most proficient method to appropriately clean and sterilize breast pumps. [67].

In the case of isolation, the mother is urged to express her breastmilk, with the help of another person to feed the baby [58]. Even though the child would not be breastfed, mothers should still wash their hands before and after pumping [68]. Walker et al. [69] collected data on the type of birth and isolated babies and babies with their mothers. He observed that babies born by vaginal birth and with their mothers had low corona-positive rate than babies born by caesarian and isolated.

## Instructions to be followed for mothers who choose to pump:

- The counter or tabletop surface utilized while pumping should be cleaned with disinfecting products with an alcohol percentage from 60 to 90%.
- The interior and exterior of the pump must be cleaned following the manufacturer's instructions as written, before and after pumping.
- The pump pack can be cleaned by a dishwasher if it is dishwasher-safe.
- Pump parts must be sanitized a minimum of once daily following instructions using steam, boiling for about five minutes, or in a dishwasher with a clean and sanitized setting.
- Pump parts must never be set in the sink and must be cleaned as quickly as time permits after the pumping.
- Thoroughly clean the washbowl and the brush (whenever utilized) with soap and water after each use, and let air dry [70]
- In case the mother is too unwell to proceed with breastfeeding, or even expressing milk, she is advised to consider re-lactation (Restarting breastfeeding after a break), wet nursing (having another lady breastfeeding the child), utilizing donor human milk, and keeping powdered milk as the last resort. The woman should speak to her doctor and find a solution based on her culture, convenience, and availability of resources [71].

### Importance of skilled counseling services on breastfeeding and outcomes

Breastfeeding counseling is a conversation in which someone with adequate training interacts and responds to the thoughts and feelings of a woman regarding breastfeeding and offers advice for her and the baby's benefit. Breastfeeding counseling provides education, reassurance, practical and problem-solving skills, and anticipatory guidance in periods of pregnancy, birth, and postpartum (around 2 months). It is open, affordable, and given to all breastfeeding mothers except in emergencies and humanitarian crises [72].

During the pandemic, the Ministry of Health and Prevention (MOHAP) set up a national call center and made a repository of all the lactation consultants with their contact numbers, to enable lactating mothers with any breastfeeding difficulties to reach out directly to their lactation consultants, avoiding hospitals visits and addressing their concerns by phone, especially during the weekends. This step was highly applauded by the local population and was a successful mother and baby-friendly initiative from MOHAP, UAE.

## Innovative solutions for breastfeeding and the ministry of health and prevention (MOHAP) UAE experience

There is abundant evidence that breastfeeding reduces the risk of babies developing infectious diseases as there are live constituents in human milk including immunoglobulins, antiviral factors, cytokines, and leukocytes that help destroy harmful pathogens. The decision on whether to keep the baby with the mother or not should be based on proper counseling and after a proper discussion about the advantages and disadvantages. The decision to keep the baby with the mother should be based on:

- The mother’s ability to safely take care of her baby
- Her capability and judgment on when and how to access the healthcare system or urgent/emergent conditions
- Her psychological wellbeing
- The availability of a healthy family member in case the mother does not want to keep the baby with her

### Role of the lactation consultant for COVID-19 patients with mild symptoms only:

- The physician lactation consultant will call the mother or the pregnant woman.
- Educate the mother about breastfeeding lessons during COVID-19, safe breastfeeding practices, safe pumping, and milk transportation
- Enquire about any issues related to breastfeeding.
- Provide support to pregnant women and breastfeeding mother

### Educational materials for pregnant women (lessons 1–3), and mothers (Mothers’ leaflets based on the individual case) sent through email:

- Directly to the mother
- Or to the nurse in the isolation building (if a printer is available she can then give a hard copy to the mother)

N-B: If the mother has no email or there is no printer in the isolation building, consider any other feasible option(s): e.g., sending hard copies with the driver, using WhatsApp…

### Use of pump in isolation building

- Preferably hospital-grade pumps (e.g., Lactina), with the corresponding pumping sets (preferably disposable)
- Otherwise, the mother can use an electric double pump

Alternate solutions may be considered when conducting lactation support services, such as telemedicine. Lactation service providers providing in-person contact with suspected or confirmed COVID-19 mothers/infants should follow recommended infection prevention and control steps, including the use of appropriate personal protective equipment (PPE).

If no PPE is available, lactation service providers will carefully consider whether other methods would mitigate the lactation service provider's risk of infection and be healthy for breastfeeding mothers and childcare and financial distress caused by income loss.

### Message for mothers who defer breastfeeding due to fear of transmitting COVID-19 via breast milk

- The anxiety of a mother or family about COVID-19 should be acknowledged as part of counseling and answered with the following messages:
- Breastfeeding and skin-to-skin contact considerably reduce the risk of death in newborns and young children and provide immediate and lifelong health and development advantages. Breastfeeding also reduces the risk of breast and ovarian cancer for the mother.
- Newborns and infants are at minimal risk of COVID-19 infection. Among the few confirmed cases of infection with COVID-19 in young children, the majority experienced only mild or asymptomatic illness.
- The benefits of breastfeeding greatly outweigh the possible consequences of COVID-19-induced infection and illness.
- Active COVID-19 has not been detected in any mother's breast milk with a confirmed suspected COVID-19 and there is no evidence to date that the virus is transmitted through breastfeeding [73]

## Conclusion

Vaccines provide individual as well as community-level protection against the emerging novel variants, as they elicit a broad immune response involving antibodies and T-cells. The protection is not only limited to mothers but also transferred to their newborns through antibodies passing through breast milk.

Breastfeeding is paramount for the overall health of both the mother and the child as it encourages bonding between the two, and reduces the risk of diseases such as diabetes, Crohn’s disease, and childhood cancers. Therefore, breastfeeding may not be ceased even in case of COVID-19 infection, flu, or other viral infection. We believe that with proper adherence to CDC hygiene recommendations, breastfeeding should be continued and encouraged. After a risk–benefit analysis, it is evident that the benefits of breastfeeding greatly outweigh the risks. Maternity centers, nursing homes, and healthcare providers have the responsibility to spread awareness and encourage new mothers to get vaccinated and continue breastfeeding to burst the COVID-19-related myths and enhance their psychological and mental health.

## Data Availability

Comprehensive review.

## Abbreviations

COVID-19: Coronavirus
AAP: American academy of pediatrics
UAE: United Arab Emirates
SARS-COV-2: Severe acute respiratory syndrome coronavirus 2
MESH: Medical subject headings
PRISMA: Preferred reporting items for systematic reviews and meta-analyses
RAKMHSU-REC: Ras Al-Khaimah medical & health sciences university research and ethics committee
IgM: Immunoglobulin
MBC: Memory B Cells
MERS: Middle east respiratory syndrome
ORF7 gene: Open reading frame
NSP2: Non-structural protein
CEO: Chief executive officer
DHA: Dubai health authority
WHO: World health organization
CDC: Center for disease control
ISUOG: International society of ultrasound in obstetrics and gynecology
N-B: Nota bene
PPE: Personal protective equipment
XBB & BF.7: Emerging omicron variants

## References

[1] Older Adults Risks and Vaccine Information [Internet]. 2022 [cited 2022 Oct 17]. Available from: https://www.cdc.gov/aging/covid19/covid19-older-adults.html - Google Search [Internet]. [cited 2022 Oct 31]. Available from: https://www.google.com/search?q=Older+Adults+Risks+and+Vaccine+Information+%5BInternet%5D.+2022+%5Bcited+2022+Oct+17%5D.+Available+from%3A+https%3A%2F%2Fwww.cdc.gov%2Faging%2Fcovid19%2Fcovid19-older-adults.html&oq=Older+Adults+Risks+and+Vaccine+Informatio

[2] Becker GE, Zambrano P, Ching C, Cashin J, Burns A, Policarpo E (2022). Global evidence of persistent violations of the international code of marketing of breast-milk substitutes: a systematic scoping review. Matern Child Nutr.

[3] World Health Organization. (2021). WHO EMRO | Breastfeeding advice during the COVID-19 outbreak | COVID-19 | Nutrition site. https://www.emro.who.int/noncommunicable-diseases/campaigns/breastfeeding-advice-during-the-covid-19-outbreak.html

[4] Peroni DG, Fanos V (2020). Lactoferrin is an important factor when breastfeeding and COVID-19 are considered. Acta Paediatr.

[5] Hand IL, Noble L (2020). Covid-19, and breastfeeding: what’s the risk?. J Perinatol.

[6] Pang Z, Hu R, Tian L, Lou F, Chen Y, Wang S (2022). Overview of Breastfeeding Under COVID-19 Pandemic. Front Immunol.

[7] Baricelli J, Rocafull MA, Vázquez D, Bastidas B, Báez-Ramirez E, Thomas LE (2015). β-defensin-2 in breast milk displays a broad antimicrobial activity against pathogenic bacteria. J Pediatr (Rio J).

[8] Magnazi MB, Sartena G, Goldberg M, Zimmerman D, Ophir E, Baruch R (2022). Impact of the COVID-19 pandemic on breastfeeding in Israel: a cross- sectional, observational survey. Int Breastfeed J.

[9] Longueira Y, Ojeda DS, Battistelli RBA, Sanchez L, Oviedo Rouco S, Albano D (2022). SARS-CoV-2-Specific IgG and IgA response in maternal blood and breastmilk of vaccinated naïve and convalescent lactating participants. Front Immunol.

[10] Charepe N, Gonçalves J, Juliano AM, Lopes DG, Canhão H, Soares H (2021). COVID-19 mRNA vaccine and antibody response in lactating women: a prospective cohort study. BMC Pregnancy Childbirth.

[11] Vassilopoulou E, Feketea G, Koumbi L, Mesiari C, Berghea EC, Konstantinou GN (2021). Breastfeeding and COVID-19: from nutrition to immunity. Front Immunol.

[12] Admyre C, Johansson SM, Qazi KR, Filén J-J, Lahesmaa R, Norman M (2007). Exosomes with immune modulatory features are present in human breast milk. J Immunol.

[13] Carsetti R, Quintarelli C, Quinti I, Piano Mortari E, Zumla A, Ippolito G (2020). The immune system of children: the key to understanding SARS-CoV-2 susceptibility?. Lancet Child Adolesc Heal.

[14] Yu JC, Khodadadi H, Salles ÉL, Pham Q, Patel P, Baban B (2021). High levels of interferon-alpha expressing macrophages in human breast milk during SARS-CoV-2 infection: a case report. Breastfeed Med.

[15] Narayanaswamy V, Pentecost BT, Telfer JC, Burnside AS, Schneider SS, Alfandari D (2022). Durable antibody and effector memory T cell responses in breastmilk from women with SARS-CoV-2. Front Immunol.

[16] Pace RM, Williams JE, Järvinen KM, Belfort MB, Pace CDW, Lackey KA (2021). Characterization of sars-cov-2 rna, antibodies, and neutralizing capacity in milk produced by women with covid-19. MBio.

[17] Mosalli R, Paes B (2020). Setting realistic goals for feeding infants when their mothers have suspected or confirmed COVID-19. Acta Paediatr Int J Paediatr.

[18] Phan T (2020). Genetic diversity and evolution of SARS-CoV-2. Infect Genet Evol.

[19] Islam MR, Hoque MN, Rahman MS, Alam ASMRU, Akther M, Puspo JA (2020). Genome-wide analysis of SARS-CoV-2 virus strains circulating worldwide implicates heterogeneity. Sci Rep.

[20] Jauregui AR, Savalia D, Lowry VK, Farrell CM, Wathelet MG (2013). Identification of residues of SARS-CoV nsp1 that differentially affect inhibition of gene expression and antiviral signaling. PLoS ONE.

[21] Andersen KG, Rambaut A, Lipkin WI, Holmes EC, Garry RF (2020). The proximal origin of SARS-CoV-2. Nat Med.

[22] Mumcuoglu O, Mackos D, Vardon E, Liffey K 2022 Factbox: Countries making COVID-19 vaccines mandatory. Reuters. 2021

[23] Shimabukuro TT, Kim SY, Myers TR, Moro PL, Oduyebo T, Panagiotakopoulos L (2021). Preliminary findings of mRNA Covid-19 vaccine safety in pregnant persons. N Engl J Med.

[24] Low JM, Lee LY, Ng YPM, Zhong Y, Amin Z (2022). Breastfeeding mother and child clinical outcomes after COVID-19 vaccination. J Hum Lact.

[25] Muyldermans J, De Weerdt L, De Brabandere L, Maertens K, Tommelein E (2022). The effects of COVID-19 vaccination on lactating women: a systematic review of the literature. Front Immunol.

[26] Perez SE, Centeno LDL, Cheng WA, Ruiz CJM, Lee Y, Congrave-Wilson Z (2022). Human Milk SARS-CoV-2 antibodies up to 6 months after vaccination. Pediatrics.

[27] Whited N, Cervantes J (2022). Antibodies against SARS-CoV-2 in human breast milk after vaccination: a systematic review and meta-analysis. Breastfeed Med.

[28] Perl SH, Uzan-Yulzari A, Klainer H, Asiskovich L, Youngster M, Rinott E (2021). SARS-CoV-2-specific antibodies in breast milk after COVID-19 vaccination of breastfeeding women. Journal of the American Medical Association JAMA.

[29] Low JM, Low YW, Zhong Y, Lee CYC, Chan M, Ng NBH (2022). Titres and neutralising capacity of SARS-CoV-2-specific antibodies in human milk: a systematic review. Arch Dis Child Fetal Neonatal Ed.

[30] Garg I, Shekhar R, Sheikh AB, Pal S (2021). COVID-19 Vaccine in Pregnant and Lactating Women: A Review of Existing Evidence and Practice Guidelines. Infect Dis Rep..

[31] Mulleners SJ, Juncker HG, van Gils MJ, van Goudoever JB, van Keulen BJ (2022). Human milk antibody response after combining two different COVID-19 vaccines: mix-and-match. J Hum Lact.

[32] McLaurin-Jiang S, Garner CD, Krutsch K, Hale TW (2021). Maternal and child symptoms following COVID-19 vaccination among breastfeeding mothers. Breastfeed Med.

[33] Yeo KT, Chia WN, Tan CW, Ong C, Yeo JG, Zhang J (2022). Neutralizing activity and SARS-CoV-2 vaccine mrna persistence in serum and breastmilk after BNT162b2 vaccination in lactating women. Front Immunol.

[34] Selma-Royo M, Bäuerl C, Mena-Tudela D, Aguilar-Camprubí L, Pérez-Cano FJ, Parra-Llorca A (2022). Anti-SARS-CoV-2 IgA and IgG in human milk after vaccination is dependent on vaccine type and previous SARS-CoV-2 exposure: a longitudinal study. Genome Med.

[35] Centers for Disease Control and Prevention. Understanding How COVID-19 Vaccines Work|CDC. Cdc.Gov. 2022 [cited 2022 Oct 31]. Available from: https://www.cdc.gov/coronavirus/2019-ncov/vaccines/different-vaccines/how-they-work.html

[36] Davanzo R, Agosti M, Cetin I, Chiantera A, Corsello G, Ramenghi LA (2021). Breastfeeding and COVID-19 vaccination: position statement of the Italian scientific societies. Ital J Pediatr.

[37] Lechosa-Muñiz C, Paz-Zulueta M, Mendez-Legaza JM, Irure-Ventura J, González RC, Montes JC (2021). Induction of sars-cov-2-specific igg and iga in serum and milk with different sars-cov-2 vaccines in breastfeeding women: a cross-sectional study in northern spain. Int J Environ Res Public Health.

[38] Vaccinations | Breastfeeding | CDC. (2022) Available from: https://www.cdc.gov/breastfeeding/breastfeeding-special-circumstances/vaccinations-medications-drugs/vaccinations.html

[39] Bertrand K, Honerkamp-Smith G, Chambers CD (2021). Maternal and Child Outcomes Reported by Breastfeeding Women Following Messenger RNA COVID-19 Vaccination. Breastfeed Med.

[40] Martins I, Louwen F, Ayres-de- Campos D, Mahmood T (2021). EBCOG position statement on COVID-19 vaccination for pregnant and breastfeeding women. Eur J Obstet Gynecol Reprod Biol.

[41] Lamers M, Van Der Mijle A, Van Hunsel F, De Vries L, Van Puijenbroek E, Ceulemans M (2022). COVID-19 vaccination during breastfeeding and its possible negative effect on milk production and supply: a preliminary observation. Breastfeed Med.

[42] Olearo F, Radmanesh LS, Felber N, von Possel R, Emmerich P, Pekarek N (2022). Anti-SARS-CoV-2 antibodies in breast milk during lactation after infection or vaccination: A cohort study. J Reprod Immunol.

[43] World Health Organization Recommendations for national [Internet]. [cited 2022 Oct 31]. Available from: https://birthdefects.org/covid-19-pregnancy-childbirth-breastfeeding/?gclid=CjwKCAjw5P2aBhAlEiwAAdY7dFIFUgGJSbK-yKnVL4PsRpNw3XPPndfpO67Pm8OKMqGpE2gDNfauIxoCMnoQAvD_BwE

[44] (CDC) C for DC and P. Coronavirus Disease (COVID-19) and Breastfeeding | Breastfeeding | CDC [Internet]. 2020 [cited 2022 Oct 31]. Available from: https://www.cdc.gov/breastfeeding/breastfeeding-special-circumstances/maternal-or-infant-illnesses/covid-19-and-breastfeeding.html

[45] Wu Y, Liu C, Dong L, Zhang C, Chen Y, Liu J (2020). Coronavirus disease 2019 among pregnant Chinese women: case series data on the safety of vaginal birth and breastfeeding. BJOG An Int J Obstet Gynaecol.

[46] Groß R, Conzelmann C, Müller JA, Stenger S, Steinhart K, Kirchhoff F (2020). Detection of SARS-CoV-2 in human breastmilk. The Lancet.

[47] World Health Organization. New FAQs address healthcare workers questions on breastfeeding and COVID-19 [Internet]. 2020 [cited 2022 Oct 31]. Available from: https://www.who.int/news/item/28-04-2020-new-faqs-address-healthcare-workers-questions-on-breastfeeding-and-covid-19

[48] Galindo-Sevilla NDC, Contreras-Carreto NA, Rojas-Bernabé A, Mancilla-Ramírez J (2021). Breastfeeding and covid-19. Gac Med Mex.

[49] Hoff CE, Movva N, Rosen Vollmar AK, Pérez-Escamilla R (2019). Impact of maternal anxiety on breastfeeding outcomes: a systematic review. Adv Nutr.

[50] Spatz DL, Davanzo R, Müller JA, Powell R, Rigourd V, Yates A (2021). Promoting and protecting human milk and breastfeeding in a COVID-19 world. Front Pediatr.

[51] Lawrence RM: Transmission of infectious diseases through breast milk and breastfeeding. In: Breastfeeding. Elsevier. Pp. 406–473 (2011)

[52] Pereira A, Cruz-Melguizo S, Adrien M, Fuentes L, Marin E, Forti A (2020). Breastfeeding mothers with COVID-19 infection: A case series. Int Breastfeed J.

[53] Rosenberg-Friedman M, Kigel A, Bahar Y, Werbner M, Alter J, Yogev Y (2021). BNT162b2 mRNA vaccine elicited antibody response in blood and milk of breastfeeding women. Nat Commun.

[54] Li F, Feng ZC, Shi Y (2020). Proposal for prevention and control of the 2019 novel coronavirus disease in newborn infants, archives of disease in childhood: fetal and neonatal edition. Arch Dis Child Fetal Neonatal Ed.

[55] Wang W, Xu Y, Gao R, Lu R, Han K, Wu G (2020). Detection of SARS-CoV-2 in Different Types of Clinical Specimens. JAMA.

[56] Favre G, Pomar L, Qi X, Nielsen-Saines K, Musso D, Baud D (2020). Guidelines for pregnant women with suspected SARS-CoV-2 infection. Lancet Infect Dis.

[57] Wang L, Shi Y, Xiao T, Fu J, Feng X, Mu D (2020). Chinese expert consensus on the perinatal and neonatal management for the prevention and control of the 2019 novel coronavirus infection (First edition). Ann Transl Med.

[58] Lubbe W, Botha E, Niela-Vilen H, Reimers P (2020). Breastfeeding during the COVID-19 pandemic–a literature review for clinical practice. Int Breastfeed J.

[59] Abuidhail J, Tamim F, Abdelrahman RY, Al-Shalabi E (2022). Knowledge and practices of breastfeeding mothers towards prevention of the emerging corona virus (COVID- 19). Glob Pediatr..

[60] Davanzo R (2020). Breast feeding at the time of COVID-19: Do not forget expressed mother’s milk, please. Arch Dis Child Fetal Neonatal Ed.

[61] Moro GE, Bertino E (2020). Breastfeeding, human milk collection and containers, and human milk banking: hot topics during the COVID-19 pandemic. J Hum Lact.

[62] Marinelli KA, Lawrence RM (2020). Safe handling of containers of expressed human milk in all settings during the SARS-CoV-2 (COVID-19) pandemic. J Hum Lact.

[63] Breastfeeding During the COVID-19 Pandemic. Pediatr Patient Educ (2021). Available from: https://publications.aap.org/patiented/article/doi/10.1542/ppe_document201/418/Breastfeeding-During-the-COVID-19-Pandemic

[64] Davanzo R, Moro G, Sandri F, Agosti M, Moretti C, Mosca F (2020). Breastfeeding and coronavirus disease-2019: Ad interim indications of the Italian Society of Neonatology endorsed by the Union of European Neonatal & Perinatal Societies. Matern Child Nutr.

[65] Breastfeeding is recommended during pandemic, but coronavirus has changed support systems [Internet] (2022). Available from: https://theconversation.com/breastfeeding-is-recommended-during-pandemic-but-coronavirus-has-changed-support-systems-136806

[66] Turner S, McGann B, Merilee BM (2022). A review of the disruption of breastfeeding supports in response to the COVID-19 pandemic in five Western countries and applications for clinical practice. Int Breastfeed J.

[67] ABM STATEMENT CORONAVIRUS. [Cited 2022 Oct 31]; Available from: https://www.bfmed.org/abm-statement-coronavirus

[68] Centers for Disease Control and Prevention. Breastfeeding and Caring for Newborns if You Have COVID-19 [Internet]. COVID-19. 2022 [cited 2022 Oct 31]. Available from: https://www.cdc.gov/coronavirus/2019-ncov/if-you-are-sick/pregnancy-breastfeeding.html

[69] Walker KF, O’Donoghue K, Grace N, Dorling J, Comeau JL, Li W (2020). Maternal transmission of SARS-COV-2 to the neonate, and possible routes for such transmission: a systematic review and critical analysis. BJOG.

[70] Breastfeeding with Coronavirus. Johns Hopkins Medicine (2022). Available from: https://www.hopkinsmedicine.org/health/conditions-and-diseases/coronavirus/breastfeeding-with-coronavirus

[71] UNICEF. Pregnancy, breastfeeding and coronavirus [Internet]. UNICEF. 2019 [cited 2022 Oct 31]. Available from: https://www.unicef.org/serbia/en/pregnancy-breastfeeding-and-coronavirus

[72] World Health Organization. WHO EMRO Breastfeeding advice during the COVID-19 outbreak COVID-19 Nutrition site 2021. Cited 2022 Oct 31. Available from: https://www.emro.who.int/noncommunicable-diseases/campaigns/breastfeeding-advice-during-the-covid-19-outbreak.html

[73] Vazquez-Vazquez A, Dib S, Rougeaux E, Wells JC, Fewtrell MS (2021). The impact of the Covid-19 lockdown on the experiences and feeding practices of new mothers in the UK: Preliminary data from the COVID-19 New Mum Study. Appetite.

